# Feasibility and acceptability of a guided internet-based stress management intervention for university students with high levels of stress: Protocol for an open trial

**DOI:** 10.1016/j.invent.2021.100369

**Published:** 2021-02-03

**Authors:** Yagmur Amanvermez, Eirini Karyotaki, Pim Cuijpers, Elske Salemink, Philip Spinhoven, Sascha Struijs, Leonore M. de Wit

**Affiliations:** a Department of Clinical, Neuro and Developmental Psychology, Amsterdam Public Health Research Institute, Vrije Universiteit Amsterdam, the Netherlands; b Department of Clinical Psychology, Utrecht University, Utrecht, the Netherlands; c Institute of Psychology, Leiden University, Leiden, the Netherlands; d Department of Psychiatry, Leiden University Medical Center, Leiden, the Netherlands

**Keywords:** Stress management, College students, Internet-based interventions, Feasibility study

## Abstract

More than half of university students have high levels of stress. Stress management programs can help students improve coping skills and prevent psychological distress. However, studies have generally targeted all university students regardless of whether they experience high levels of stress or not, and thus more studies are needed to examine the feasibility and acceptability of e-health interventions for students with elevated stress. The present open trial aims to examine the feasibility and acceptability of a guided internet-based stress management program for university students with high levels of stress. In this study, participants are recruited via e-mail, newsletters, and flyers from four universities in the Netherlands to participate in a guided internet-based stress management program. Guidance is delivered by e-coaches who provide weekly asynchronous text-based motivational feedback after each module is completed. Primary outcomes are satisfaction with the intervention, assessed by the Client Satisfaction Scale (CSQ-8), and usability, assessed by the System Usability Scale (SUS-10). Secondary outcomes are perceived stress, quality of life, and depression, assessed by the Perceived Stress Scale (PSS-10), the EuroQol- 5 Dimension- 5 Level Scale (EQ- 5D- 5L), and the Patient Health Questionnaire (PHQ-9) respectively. Adherence rates to the program are assessed by examining the number of completed modules, time spent on the platform, and completed exercises. The Caring Universities Project was funded in (September 2019). In June 2020, the project was officially announced to the students and recruitment began immediately. As of October 2020, recruitment continues. The expected date of the publication of the results is in 2021. It is expected that the results of the proposed study will be informative for designing and implementing e-health interventions in higher education. Moreover, it is assumed that the findings will contribute to the growing literature on internet interventions by yielding preliminary evidence related to the feasibility and acceptability of an online stress management program.

**Trial registration:**

Netherlands Trial Register NL8686; https://www.trialregister.nl/trial/8686

## Highlights

• Online stress management programs can be successful to improve coping skills.
• University students face many stressors. Stressors can be associated with poor mental health and academic achievement when not being handled effectively.
• Online interventions especially targeting students with high-stress levels are limited.
• Feasibility studies are needed to provide the most appropriate stress management programs for university students at risk.

## 1 Introduction

College years coincide with the developmental phase of emerging adulthood. In this period, individuals experience several life transitions such as gaining autonomy, changing social roles, and new responsibilities (Sussman and Arnett, 2014; Rickwood et al., 2005). The first onset of several psychological problems often occurs in this period of life (Kessler et al., 2007). Besides the positive experiences, a considerable number of university students experience several stressors related to academic demands, interpersonal problems, changing life-styles, and financial difficulties (Pierceall and Keim, 2007; Robotham, 2008). Studies have shown that 63–73% of college students report high stress levels (Leppink et al., 2016; Stallman, 2010; Saleh et al., 2017).

Although stressors can be a facilitator of motivation and increased performance (Strack et al., 2017), chronic stress is associated with the onset of mental health problems including sleeping difficulties (Amaral et al., 2018), depression (Vrshek-Schallhorn et al., 2015; Newcomb-Anjo et al., 2017), anxiety (Bergdahl and Bergdahl, 2002; Young and Dietrich, 2015), eating disorders (Torres and Nowson, 2007), substance use (Lijffijt et al., 2014), and suicide (Liu et al., 2019). Having high levels of stress can also be related to role impairment (Alonso et al., 2018), lower academic achievement (Shields, 2001), and dropout from university (Eisenberg et al., 2009; Pritchard and Wilson, 2003).

Stressors related to university life (e.g. academic demands, lifestyle changes, living away from home, etc.) are inevitable and common to almost all students. However, some students might need some form of support to be able to effectively cope with the stressors. Evidence-based interventions can help college students to acquire effective coping strategies and change their maladaptive reactions toward stressors (Regehr et al., 2013). Recent studies in young adults showed that stress management programs can reduce psychological distress (Kim et al., 2016), sleep problems (Dvořáková et al., 2017), depression (Breedvelt et al., 2018), and anxiety (Brennan et al., 2016).

Although college students experience high levels of stress, the utilization of campus mental health services is remarkably low due to several barriers (Ebert et al., 2019; Rosenthal and Wilson, 2008). Lack of perceived need for receiving help, preference for self-help, perceived stigma, negative attitudes toward the treatment, lack of time, and not being aware of the services at the campus are among the most important treatment barriers (Czyz et al., 2013; Eisenberg et al., 2007). In addition, cultural differences and language barriers were among the main reasons reported for the low uptake of on-campus counseling services by international students (Eisenberg et al., 2007; Hyun et al., 2007; Mori, 2000). Apart from the attitudinal barriers, system-related circumstances can also limit access to mental health support; these include long waiting lists and insufficient resources to meet the increased demands on counseling centers at universities (Watkins et al., 2012).

E-health interventions can be helpful in overcoming some of the aforementioned treatment barriers. The internet is used by college students for various purposes, including maintaining contact with friends or family members, building a social network, getting acquainted with the new university environment, educational reasons (Gray et al., 2010; Sleeman et al., 2016), and also for seeking health information (Horgan and Sweeney, 2010). Internet-based interventions could, therefore, be easily adopted by this population. Besides, internet interventions have additional benefits such as anonymity, flexibility in time, and easy, low-cost access to mental health support (Andersson and Titov, 2014; Ebert et al., 2017; Stallman and Kavanagh, 2018). Universities can also alleviate the growing demand for university counseling centers by offering internet interventions to their students.

Recently, studies on internet interventions have yielded promising results for a wide range of psychological complaints such as stress (Davies et al., 2014; Heber et al., 2017; Frazier et al., 2015; Heber et al., 2016), depression, and anxiety (Reyes-Portillo et al., 2014). However, only a limited number of interventions were designed according to the college students' needs. Given the specific sources of stress during the college years and the increased individual and societal benefits of maximizing the potential of college students, more attention should be given to the development and implementation of stress management programs in higher education. Moreover, existing stress management programs did not specifically target college students with high-stress levels. Although programs targeting general college student populations without screening for the problem (universal prevention) are essential to promote well-being in higher education (Conley et al., 2013), recent meta-analytic evidence showed that internet-based interventions targeting students at risk (indicated prevention) yielded larger effect sizes than those based on universal prevention (Conley et al., 2016; Harrer et al., 2019). Yet indicated prevention in this field has not been extensively investigated (Amanvermez et al., 2020). As a result, it is essential to examine the feasibility and acceptability of stress management interventions for college students with high levels of stress. Therefore, this study aims to evaluate the feasibility and acceptability of a newly developed internet-based stress management program for university students with elevated levels of stress. The secondary goal is to investigate the differences from pre-test to post-test for stress, depression, and quality of life. We also aim to examine the adherence rate of college students.

## 2 Methods

### 2.1 Study design

This study is an open trial of a guided internet-based stress management program. It employs a single-group pre- (t0) and post-test (t1) design. Five weeks after t0, t1 is administered. The present study is part of the World Health Organization (WHO) World Mental Health International College Student Initiative (WMH-ICS) (Cuijpers et al., 2019). The study protocol was approved by the Scientific and Ethical Review Board of all participating universities (Vrije University Amsterdam, Leiden University, Maastricht University, and Utrecht University). This study was also registered at the Netherlands Trial Register (Registration Number: NL8686).

### 2.2 Study setting

The Caring Universities Project is being conducted in the four above-mentioned universities in the Netherlands.

### 2.3 Participants

Participants are undergraduate or graduate students enrolled in the above-mentioned universities. Participants are screened in terms of their stress levels. According to the screening results, we include students with elevated stress levels based on the Perceived Stress Scale 10-item (PSS-10) (Cohen and Williamson, 1988a). We aim to recruit 50 students.

### 2.4 Eligibility criteria

We include participants if they meet the following criteria: 1) aged 16 or older, 2) having enrolled as bachelor, master, or Ph.D. student, 3) having elevated levels of perceived stress (PSS-10 ≥ 20.4) at screening, 4) having given informed consent. Both domestic and international students are eligible to participate in the study.

There is no established cut-off score for the PSS-10. Following the procedures of previous studies in this field, we decided to use 20.4 as a cut-off score in our study calculating the one standard deviation (*SD* = 6.2) above the mean score of 14.2 which has been found in a general population sample between 18 and 29 years old (Cohen and Williamson, 1988b). Recent findings on the PSS-10 also showed similar descriptive findings for college students (Leppink et al., 2016; Klein et al., 2016).

Exclusion criteria are 1) self-reported severe symptoms of depression (as defined by a score higher than 20 on the Patient Health Questionnaire (PHQ-9) (Kroenke et al., 2001)) or 2) experiencing suicidal risk (as defined by a score of 2 or above on the PHQ-9 item nine or a score of 1 or above for the question “In approximately how many months during the past 12 did you think about how you might kill yourself or work out a plan of how to kill yourself?” or responding somewhat likely/very likely to the question of “How likely do you think it is that you will act on this plan in the next 12 months?”).

### 2.5 Intervention

The guided internet-based stress management program (Rel@x) was developed by the authors on the basis of existing stress management techniques, and co-created with the university students to meet the specific needs of the students. We have conducted several focus group discussions and interviews with the students to understand their opinions about this program and their needs. We have tailored the program to align with their preferences. The stress management program is based on cognitive-behavioral therapy (CBT) and Lazarus and Folkman's transactional model of stress (Lazarus and Folkman, 1984). It comprises five modules that are delivered via computer, laptop, tablet, or mobile phone. Each module takes approximately 60 min to complete. Students will be advised to complete one module per week. Accordingly, the duration of the stress management program is approximately five weeks. However, participants can follow the program at their own pace. Every module consists of evidence-based information, exercises, fictitious examples, and homework assignments. The content is delivered in text format with pictures and infographics; some modules include video clips and audio recordings on a relevant subject. The intervention is available in both English and Dutch.

Modules cover information about stress, coping strategies, cognitive restructuring, and problem-solving skills. At the beginning of the intervention, an introduction module is provided. This introduction module gives general information about the use of the platform and the basics of the modules to set realistic intervention expectations. The first module covers information about stress, exercises about identifying the stressors and symptoms of stress, and the consequences of high levels of stress on health. The second module introduces the coping strategies (i.e. emotion-based coping, problem-based coping, and meaning-based coping) and the effect of perceived control over stressful situations. The third module includes psychoeducation on cognitive restructuring and several exercises to identify and challenge negative thinking patterns. In the fourth module, problem-solving steps are explained with the exercises. The last module includes a summary of each module, exercises to review the participant's progress, and setting goals for the future. Several tips are provided to transfer the information and strategies from the modules into everyday life.

Each module begins with a review of the homework assignment from the previous module and ends with a summary of the module. In addition to the main modules, there are four optional modules which students can select based on their needs. The topics of the optional modules are 1) time management and procrastination, 2) assertiveness, 3) adaptation to a new culture, 4) sleep, emotional eating, and exercise. The optional modules are available at the end of each module.

### 2.6 E-coaches

Participants receive feedback from the e-coaches after completing each module. E-coaches are (research) master students in clinical psychology and 3rd-year clinical psychology bachelor students (at the end of the 2nd semester) who meet the criteria of specific courses namely 1) coaching and assessment, 2) low-intensity treatments for common mental health problems, and 3) diversity in clinical practice. The e-coaches were selected in the following way: After attending an informative meeting, interested students responded in writing, outlining their motivation to be an e-coach in this project. Selected students were invited for an interview with the research team. After completing the selection, students completed 5 1/2 hours of training including a supervised intervision meeting during which the e-coaches discuss their feedback in a plenary session. These e-coaches are regularly supervised by the research team, including a clinical psychologist, for the duration of the intervention. E-coaches will provide asynchronous written personalized feedback to each participant via the intervention platform. The written feedback aims to increase motivation and adherence. Participants also receive automatic reminders to increase engagement in cases of two weeks of inactivity.

### 2.7 Platform

The intervention is delivered via the Caring Universities platform. This platform has been developed for e-health applications for college students in the Caring Universities project and includes other interventions on several issues for college students. On this platform, researchers can add and arrange intervention content, materials (e.g. video, visuals), and questionnaires. Students and e-coaches can create an account and log on to the platform with a user name and self-generated password via laptops, tablets, and mobile phones. This platform is accessible 24/7 for the participants. After completing the intervention and outcome assessments, participants will still be able to access the platform. A stress journal and a single-item question to keep track of stress levels were integrated into the platform for self-monitoring. Moreover, the e-coach and student can send text messages to each other on this platform. The platform complies with the General Data Protection Regulation (GDPR).

### 2.8 Assessment measures

#### 2.8.1 Primary outcomes

##### 2.8.1.1 Satisfaction with the intervention

Participants' satisfaction with the intervention is assessed by the Client Satisfaction Questionnaire (CSQ-8) (Larsen et al., 1979). This self-report questionnaire has been widely used to measure satisfaction levels with an online intervention. This questionnaire includes eight items on a 4-point scale. The total score ranges from 8 to 32 and a higher score indicates greater satisfaction. The psychometric properties of the scale showed high reliability and validity for the web-based interventions (Boß et al., 2016). The Dutch translation of the CSQ-8 also showed similar properties (De Brey, 1983).

##### 2.8.1.2 Usability

The usability of the intervention is measured by the System Usability Scale (SUS-10) (Brooke, 1996). This scale consists of 10 items on a 5-point Likert scale. The total score can range from 0 to 100. Previous studies examining the reliability and validity of the scale showed promising results (Lewis, 2018). Although the Dutch translation of the SUS-10 has been used in previous studies (Wever et al., 2012), published psychometric properties of the scale in Dutch could not be retrieved. However, a comprehensive review of all studies using this scale indicated adequate reliability (Bangor et al., 2008).

#### 2.8.2 Secondary outcomes

Secondary outcomes of this study are perceived stress, depression, quality of life, and adherence.

##### 2.8.2.1 Perceived stress

The Perceived Stress Scale (PSS-10) is used to assess the stress levels (Cohen and Williamson, 1988a; Cohen et al., 1983). This scale is a brief, easy-to-use self-report scale containing 10 items on a 5-point Likert scale. The scale aims to assess the extent to which participants evaluate their life as unpredictable and uncontrollable. Higher scores indicate higher perceived stress. The PSS-10 is used to assess the changes from pre- to post-scores. In this study, we have also added the short version of the PSS-10 to track the stress levels of the participants on a weekly basis. The short version of this scale includes four items from the PSS-10. The PSS-4 is sent to the students to gain a deeper understanding of the process of change. The PSS-10 and the PSS-4 showed high reliability and validity (Cohen et al., 1983; Lee, 2012). The PSS yielded good psychometrics in college students (Denovan et al., 2019).

##### 2.8.2.2 Depressive symptoms

The PHQ-9 is used to assess depression (Kroenke et al., 2001). Each item is rated on a 0–3 scale. Higher scores indicate a higher level of depressive symptoms. The scores of 1–4, 5–9, 10–14, 15–19, and 20–27 indicate no depression, mild depression, moderate depression, moderately severe depression, and severe depression, respectively (Kroenke et al., 2001).

##### 2.8.2.3 Quality of life

Quality of life is measured by the EuroQol-5 Dimension-5 Level Scale (EQ-5D-5L) (Group TE, 1990). This scale has five dimensions namely mobility, self-care, usual activities, pain/discomfort, and anxiety/depression, with each dimension consisting of five levels indicating the severity of the problem. Psychometric studies of the EQ-5D-5L showed promising and robust results in several patient samples (Janssen et al., 2013; Herdman et al., 2011).

##### 2.8.2.4 Adherence

Adherence is defined as the extent to which the individuals get involved in the intervention (Christensen et al., 2009). In the present study, adherence is measured primarily by the total number of modules completed by each participant at the post-test. The number of completed modules is divided by the total number of modules to calculate the completion rate. Moreover, we will assess other indicators of adherence, such as the time spent in the intervention, the number of logins, and the number of completed exercises.

#### 2.8.3 Additional measures

##### 2.8.3.1 Sociodemographic information

In order to examine the characteristics of the sample, sociodemographic information is collected such as age, gender, ethnicity, university, faculty, marital status, and whether they are receiving psychological treatment (psychotherapy or medication).

##### 2.8.3.2 E-coach evaluation

We also ask participants to evaluate the e-coach using the Working Alliance Inventory for guided internet interventions (WAI-I) (Martín et al., 2020). This inventory contains 12 items on a 5-point Likert scale with two dimensions: task & goal agreement with the therapist and bond with the therapist. The WAI-I measures the perceived collaboration with the therapist from the perspective of the patient. The psychometric characteristics of the WAI-I yielded adequate results (Martín et al., 2020).

##### 2.8.3.3 Evaluation of each module

After completing each module, participants are asked to give feedback about the appropriateness of the modules for the college student sample. The questions about the evaluation of the modules were derived from another study (Rahmadiana et al., 2019). Participants are asked the following questions: 1) Were the goals of this Internet module clearly defined? 2) Was the content in this module clear and easy to understand? 3) Was this module easy to navigate? 4) Was the length of this module appropriate to the topic? 5) Were the illustrated pictures in this module helpful? 6) Did you understand the language, idiom, and words used in this module? 7) Do you think the case examples given in this module were appropriate for university students? We also ask three open-ended questions as follows: 1) What did you learn from this module? 2) Is there anything you would want to change in this module? 3) Is there anything you want to mention about this module?

Moreover, if feasible, we will explore possible stressors based on the answers of participants in the questions and exercises of the intervention.

#### 2.8.4 Semi-structured interviews

We conduct in-depth interviews with a selection of at least 10 participants among the students who complete all main modules. The selection of the participants is based on the maximum variation sampling, taking into account equal representation of gender groups, varying levels of satisfaction with the program (CSQ-8), usability (SUS-10), and the changed scores of perceived stress (PSS-10) from pre-test to post-test. We select some students showing little or no improvement and some students with greater improvement in perceived stress. Similarly, we select a few students who show low satisfaction and a few students with high satisfaction with the program. Semi-structured interviews are conducted with a set of questions based on previous studies to examine the user experiences in a newly developed Internet-based intervention (Devi et al., 2014; Fleischmann et al., 2018). The original questions have been revised to fit the purpose of our study: 1) What were your initial thoughts and feelings regarding this Internet-based stress program? 2) Why did you prefer this program as an internet-based treatment over face-to-face support? 3) How was your overall experience of using the program? 4) Do you feel anything has changed in your life since using the program? 5) Generally, how did you feel about this program? 6) To what extent did you accept the program and why? 7) Were there any enjoyable parts in the program? (And why?) 8) What was your experience of the program being online and delivered via the internet? 9) How did the program fit in with your lifestyle? 10) Is there anything you would want to change or add to this program?

#### 2.8.5 Assessments

Overview of the assessments can be seen in Table 1.

**Table 1:** Measures and assessment points.

	Assessment points		
	T0	Tx	T1
Socio-demographics	X		
Client Satisfaction Questionnaire (CSQ-8)			X
System Usability Scale (SUS-10)			X
Perceived Stress Scale (PSS-10)	X		X
Quality of life (EQ-5D-5L)	X		X
Perceived Stress Scale (PSS-4)		X	
Patient Health Questionnaire (PHQ-9)	X		X

T0 = Pre-test.

Tx = Weekly assessments.

T1 = Post-test (5-week).

### 2.9 Sample size

There is no consensus on how to calculate the sample size of an open feasibility study. Some studies suggest a sample of at least 12 participants (Julious, 2005; Moore et al., 2011) while others recommend 35 or more participants (Teare et al., 2014). Thus, in the present study, no power calculation was conducted to determine the sample size, as we mainly focus on the feasibility and acceptability of the intervention. Based on the rule of thumb and similar previous studies (Rahmadiana et al., 2019), we anticipate that 50 participants will be sufficient to examine our main objectives. For semi-structured interviews, the sample size will depend on the saturation of the codes (Sim et al., 2018). However, we aim to reach at least 10 participants, since it is well-documented that code saturation can be reached after approximately 10 interviews (Hennink et al., 2017).

### 2.10 Recruitment

Recruitment is conducted in several ways. The main recruitment strategy is the online survey of the WHO WMH-ICS. This survey includes several questionnaires measuring the mental disorders and psychological characteristics of the students to be used in epidemiological studies. The e-mail is sent out to first-year students at the Vrije University Amsterdam (VU), and all students at Leiden University, Maastricht University, and Utrecht University and includes information about the Caring Universities project and a survey link. Students receive personalized feedback after completing the survey if they wish. After administering this survey, we invite students who are eligible for inclusion in our study. Volunteer students are informed about the stress management program and asked to give informed consent. Students who do not meet the inclusion criteria will be informed about the possible sources for getting help from other relevant resources (e.g. 113 online, student psychologists, general practitioner (GP), mental health care institutes in the region). Second, we contact the International Office, student clubs, and organizations to reach students with diverse characteristics. Third, we recruit participants through student advisors, student mentors, researchers, and lecturers who can recommend this program for students who might be interested in a stress management program. Finally, we recruit students through advertisements (e.g., flyers, faculty newsletters, social media, related websites, etc.). The advertisements target all college students by informing them about the study and emphasize the importance of stress management programs in increasing well-being and improving academic performance. Students will not be compensated for participation.

### 2.11 Statistical analysis

Descriptive statistics will be calculated to examine participants' satisfaction with the intervention, usability, and adherence. We conduct two-tailed paired *t*-tests using a significance level α = 0.05 to assess changes in the scores of PSS-10, PHQ-9, and EQ- 5D5L. In addition, qualitative analysis will be conducted to examine the participants' use of the intervention, their satisfaction with the intervention, adherence, e-coach evaluation, and feedback about the modules. Moreover, semi-structured interviews with the participants will be audio-recorded and the verbal data will be transcribed. After obtaining all of the transcriptions and written responses, a thematic analysis will be conducted to identify and categorize the themes about their experiences with the intervention (Clarke & Braun, 2017). First, we will code and organize the written data under meaningful themes. After categorization of the main themes, we will examine whether they include sub-themes. At the end of the analysis, we will interpret the results based on the themes (Braun and Clarke, 2006; Howitt, 2010).

### 2.12 Possible harms

Possible harms can occur when receiving the internet-based interventions (Rozental et al., 2014). Suicide risk would be the most severe possible incident. Some students may show warning signs for suicide while participating in the stress management program. If a suicidal risk is identified by the e-coaches, the suicide prevention protocol will be applied and participation in the study will be terminated. Following this protocol, the e-coach will contact the student and evaluate the suicidal risk by conducting section C of Mini International Neuropsychiatric Interview (M.I.N.I.) (Sheehan et al., 1998). If the e-coach estimates low or moderate suicide risk, the student will be advised to get further help from a GP or 113Online which is a 24/7 accessible online platform designed for talking anonymously about suicidal thoughts. If high suicidality is indicated by the M.I.N.I., we consult with a psychiatrist about the student's specific situation. The student will be immediately referred to a GP or 113Online and closely monitored by the e-coach and the research team. Another risk of receiving this treatment can be related to deterioration (such as worsening stress symptoms), nonresponse, or occurrence of other psychological/ emotional symptoms (Rozental et al., 2014). If these negative effects should be detected, the researchers will report these findings in the manuscript. However, based on the findings of similar studies (Boettcher et al., 2014; Rozental et al., 2015), we anticipate minimal risk for the participants in this study.

## 3 Discussion

The present study aims to examine the feasibility and acceptability of an internet-based stress management program for college students with elevated levels of stress. In addition, this study will examine whether any improvements in stress levels, quality of life, and depression occur from pre-test to post-test. We anticipate that the stress management program will be perceived as feasible and acceptable by college students. Improvement in the stress, quality of life, and depression scores are also expected after receiving this program, but may not reach significance levels. Similar stress management interventions for college students showed promising results (Frazier et al., 2015; Harrer et al., 2018). However, internet-based stress management programs for pre-selected students with high levels of stress are scarce in the literature. Therefore, we believe that this study will be informative for the development and implementation of internet-based programs for at-risk students in tertiary education. In addition, stress and coping are closely associated with mental health problems such as depression and anxiety (Bergdahl and Bergdahl, 2002; Garnefski et al., 2002). Therefore designing stress management programs for college students can also be preventative for common mental health problems.

Although drop-out rates can be higher in internet-based interventions than face-to-face (Wagner et al., 2014), we have incorporated the suggested strategies from previous studies into our stress management program to increase adherence, such as adding human support (Conley et al., 2013; Mohr et al., 2011) and sending automatic reminders (Hilvert-Bruce et al., 2012). In order to maintain adherence, e-coaches will monitor the progress of participants, and send individualized reminders for the participants when they are inactive on the platform. Also in case of two weeks of inactivity, participants will receive automated reminders. Moreover, we have conducted several focus groups with college students to meet the specific needs of college students in our program. Based on this co-creation approach, we could tailor the content and the features in the platform specifically toward college students. Therefore, it is expected that this stress management program will be engaging and highly acceptable for college students. We also designed this stress management program based on the transactional theory of stress and coping by incorporating CBT components. In a recent meta-analysis, CBT-based interventions yielded a greater effect size than interventions from other theoretical backgrounds (Regehr et al., 2013; Harrer et al., 2019). Another meta-analysis also showed that CBT-based internet interventions were highly acceptable and engaging (Andrews et al., 2010). In view of the results of these studies, we expect similar results in the present study.

We also acknowledge the limitations of the present study. First, we target 50 participants for this study based on other similar work. The sample size will be informative for us to examine the feasibility and acceptability of this program; however, larger sample sizes can yield more robust results. Therefore, if we can reach more than 50 participants, we will include them in the analysis. Second, dropout can be higher in internet-based interventions. Although adding human support to the internet-based interventions was associated with lower dropout rates (Richards and Richardson, 2012), participants may discontinue the program for different reasons such as finding the program complicated, lack of time, or feeling a need for face-to-face contact (Johansson et al., 2015). Third, students will be guided by psychology students. Limitations may arise from the limited experience of the e-coaches, however, they will be supervised by researchers and clinical psychologists in this study. Fourth the generalizability of the results to non-college peers or older adults may be another limitation. Lastly, the PSS-10 and the SUS-10 have been administered in previous studies in the Netherlands. However, we could not retrieve the published psychometric characteristics of these scales in the Dutch language. In addition, the ceiling effect was reported for EQ-5D-5L in the general population (Abdin et al., 2013) and this might exist for college students in this study. Finally, we do not focus on measuring the stressors of college students in this study, as our main objective is to investigate the feasibility and acceptability of this program. Therefore, future studies are needed to explore the nature of the stressors and their associations with perceived stress in more detail.

## 4 Conclusions

Despite the limitations, internet-based stress management interventions can be feasible and acceptable for college students. We expect that the findings of the study will be informative for designing psychological interventions and developing cutting-edge mental health applications in higher education.

## Declaration of competing interest

The authors declare that they have no known competing financial interests or personal relationships that could have appeared to influence the work reported in this paper.
